# Biodegradable Cellulose-based Hydrogels: Design and Applications

**DOI:** 10.3390/ma2020353

**Published:** 2009-04-16

**Authors:** Alessandro Sannino, Christian Demitri, Marta Madaghiele

**Affiliations:** Department of Engineering for Innovation, University of Salento, Via per Monteroni, 73100 Lecce, Italy; E-Mails: christian.demitri@unile.it (C.D.); marta.madaghiele@unile.it (M.M.)

**Keywords:** Hydrogels, cellulose, biodegradation

## Abstract

Hydrogels are macromolecular networks able to absorb and release water solutions in a reversible manner, in response to specific environmental stimuli. Such stimuli-sensitive behaviour makes hydrogels appealing for the design of ‘smart’ devices, applicable in a variety of technological fields. In particular, in cases where either ecological or biocompatibility issues are concerned, the biodegradability of the hydrogel network, together with the control of the degradation rate, may provide additional value to the developed device. This review surveys the design and the applications of cellulose-based hydrogels, which are extensively investigated due to the large availability of cellulose in nature, the intrinsic degradability of cellulose and the smart behaviour displayed by some cellulose derivatives.

## 1. Introduction

Hydrophilic polymers can swell and absorb water without dissolving, provided that chemical or physical crosslinks exist among the macromolecular chains. The polymer network resulting from the crosslinks swells in the aqueous solvent, until the thermodynamic force of swelling is totally counterbalanced by the elastic, retractive force exerted by the crosslinks. This ‘solid-like solution’ of polymer and water resulting at equilibrium is known as a hydrogel. The amount of water retained by the mesh of the hydrogel network depends on the structure of the polymer network itself and on the environmental conditions, such as the temperature, pH and ionic strength of the water solution in contact with the polymer [1]. The volume or mass swelling ratio of the hydrogel is the most important variable to be evaluated for given environmental conditions, as it affects the diffusive, mechanical, optical, acoustic and surface properties of the hydrogel itself. In cases where sharp and/or fast swelling-deswelling transitions happen in response to changes of external stimuli, hydrogels are potentially useful for the development of a variety of smart devices, such as valves, artificial muscles and substrates for controlled drug release [2,3,4,5]. Since the first hydrogels based on poly(hydroxyethyl methacrylate) (PHEMA) developed by Otto Wichterle in the 1950s and later patented for use as soft contact lenses [6], great steps have been taken by researchers towards obtaining novel hydrogels, based on synthetic, natural or hybrid polymers, which possess given swelling properties and/or biocompatibility and bioactivity. Innovative hydrogel products have thus been developed either as water absorbents for specific applications (*e.g.*, personal hygiene products, underwater devices, water reservoirs for dry soils) [7,8,9,10] or as biomedical devices, including soft contact lenses, lubricating surface coatings, phantoms for ultrasound-based imaging, controlled drug release devices, wound healing dressings, cell immobilization islets, three-dimensional cell culture substrates, and bioactive scaffolds for regenerative medicine [4,5,11,12,13,14,15,16]. For many of the abovementioned applications, the biodegradation of the hydrogel is a preferred or a required design variable to be addressed. Indeed a biodurable hydrogel is neither environmentally friendly nor totally biocompatible in the long term.

This review focuses on the current design and use of cellulose-based hydrogels, which usually couple their biodegradability with a smart stimuli-sensitive behaviour. These features, together with the large availability of cellulose in nature and the low cost of cellulose derivatives, make cellulose-based hydrogels particularly attractive. In the following, after introducing the properties and the crosslinking of cellulose and its derivatives, both well-established and innovative applications of cellulose-based hydrogels are discussed, with some suggestions for future developments.

## 2. Cellulose and Cellulose-based Hydrogels

### 2.1. Cellulose Structure and Biodegradability

Cellulose is the most abundant naturally occurring polymer of glucose, found as the main constituent of plants and natural fibers such as cotton and linen. Some bacteria (*e.g.*, *Acetobacter xylinum*) are also able to synthesize cellulose [17]. Microbial or bacterial cellulose (BC) is chemically identical to plant cellulose (PC), although possessing different macromolecular structure and physical properties [18]. In both BC and PC, the glucose units are held together by 1,4-β-glucosidic linkages, which account for the high crystallinity of cellulose (usually in the range 40-60% for PC and above 60% for BC) and its insolubility in water and other common solvents. However, BC biosynthesis yields nanosized fibers, which are about two orders of magnitude smaller than PC fibers. BC cellulose thus shows a peculiar, ultrafine fiber network with high water holding capacity and superior tensile strength compared to PC. Moreover, BC is totally pure, unlike PC which is usually associated with other biogenic compounds, such as lignin and pectin. Therefore, while BC is used as synthesized by bacteria, PC requires further purification and modification. Chemical modification of cellulose, usually involving esterification or etherification of the hydroxyl groups, is performed to produce cellulose derivatives, named cellulosics, which are more easily processable and find large application in the industry. Cellulose and its derivatives are environmentally friendly, as they are degradable by several bacteria and fungi present in air, water and soil [19], which are able to synthesize cellulose-specific enzymes (*i.e.* cellulases). The biodegradation of cellulose has been widely investigated, and progressively leads to decreased molecular weight, lower mechanical strength and increased solubility. Moreover, higher biodegradation rates of cellulose are likely yielded by lower degrees of crystallinity and improved water solubility [20].

The excellent biocompatibility of cellulose, cellulosics and cellulase-mediated degradation [20,21] has prompted the large use of cellulose-based devices in biomedical applications. With regard to *in vivo* applications, it is worth reminding that cellulose is a biodurable material. Indeed, resorption of cellulose in animal and human tissues does not occur, since cells are not able to synthesize cellulases. Such a consideration points out the fundamental distinction between biodegradability and bioresorbability: the former refers to the ability of the material to be degraded by microorganisms, whereas the latter indicates the ability of the material to be digested or metabolized when implanted *in vivo*. In a pioneering long-term study by Martson *et al.* [22], a cellulose sponge implant seems to undergo a slow degradation in the rat subcutaneous tissue. However, the time length of the study (*i.e.*, 60 weeks), together with the poor observed degradation and the lack of any knowledge about the possible mechanism of *in vivo* resorption, suggest that cellulose-based implants should be considered as biodurable. Nevertheless, the chemical modification and/or crosslinking of water-soluble cellulosics with bioresorbable moieties can yield resorbable cellulose-based devices [23,24,25,26].

### 2.2. Water-soluble Cellulose Derivatives

**Figure 1:**
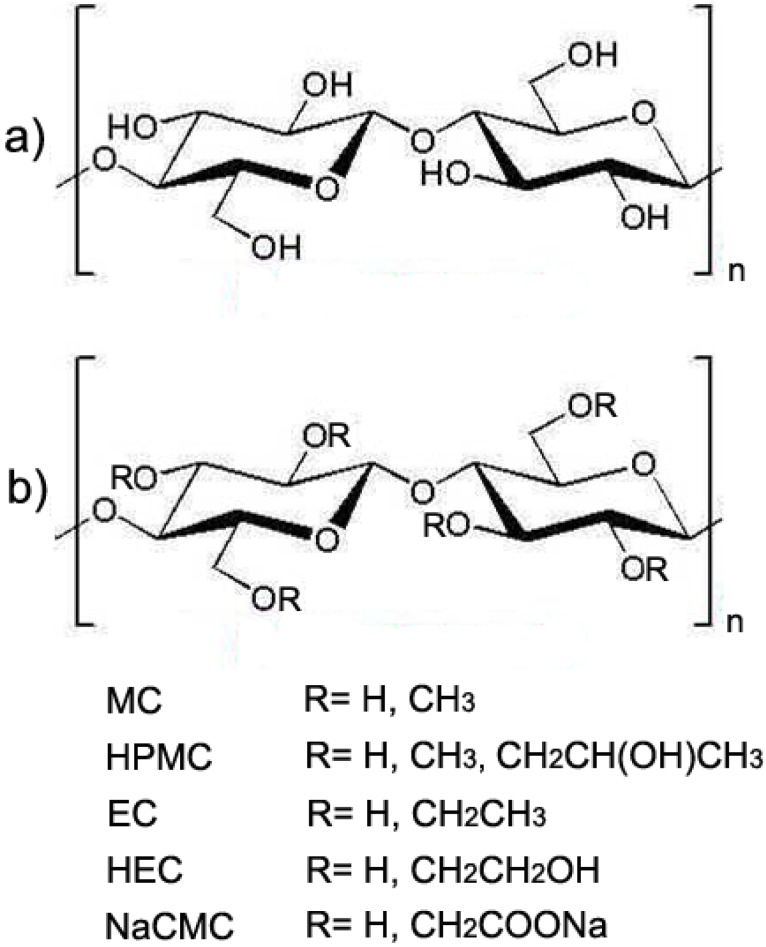
(a) Repeating unit of cellulose, also termed ‘cellubiose’. (b) Repeating unit of cellulose derivatives. The substituent group ‘R’ is indicated for methylcellulose (MC), hydroxylpropyl methylcellulose (HPMC), ethyl cellulose (EC), hydroxyethyl cellulose (HEC) and sodium carboxymethylcellulose (NaCMC).

Most water-soluble cellulose derivatives are obtained via etherification of cellulose, which involves the reaction of the hydroxyl groups of cellulose with organic species, such as methyl and ethyl units. The degree of substitution, defined as the average number of etherified hydroxyl groups in a glucose unit, can be controlled to a certain extent, in order to obtain cellulose derivatives with given solubility and viscosity in water solutions. Cellulose-based hydrogels, either reversible or stable, can be formed by properly crosslinking aqueous solutions of cellulose ethers, such as methylcellulose (MC), hydroxypropyl methylcellulose (HPMC), ethyl cellulose (EC), hydroxyethyl cellulose (HEC) and sodium carboxymethylcellulose (NaCMC), which are among the most widely used cellulose derivatives. The structure of such derivatives is shown in Figure 1. It is worth highlighting that all these polymers find wide application as thickeners and/or emulsifying agents in the food, pharmaceutical and cosmetics industries, due to their non-toxicity and low cost.

Among the abovementioned cellulose ethers, only NaCMC is a polyelectrolyte, and thus a ‘smart’ cellulose derivative which shows sensitivity to pH and ionic strength variations. Indeed the presence of NaCMC in a cellulose-based hydrogel provides the hydrogel itself with electrostatic charges anchored to the network, which have a double effect on the swelling capability. On one side, the electrostatic repulsion established between charges of the same sign forces the polymer chains to a more elongated state than that found in a neutral network, thus increasing the swelling. On the other, the counterions that are present in the gel to ensure macroscopic electrical neutrality induce more water to enter the network, due to a Donnan type effect [27]. The Donnan contribution to the osmotic pressure is dependent on the different concentration of mobile counterions between the gel and the external solution, thus making the gel sensitive to variations of pH or ionic strength. The polyelectrolyte nature of NaCMC makes it ideal for the development of superabsorbent hydrogels with a smart behaviour [28,29].

### 2.3. Cellulose-based Hydrogels and Crosslinking Strategies

Cellulose-based hydrogels can be obtained via either physical or chemical stabilization of aqueous solutions of cellulosics. Additional natural and/or synthetic polymers might be combined with cellulose to obtain composite hydrogels with specific properties [30,31]. Physical, thermoreversible gels are usually prepared from water solutions of methylcellulose and/or hydroxypropyl methylcellulose (in a concentration of 1-10% by weight) [32]. The gelation mechanism involves hydrophobic associations among the macromolecules possessing the methoxy group. At low temperatures, polymer chains in solution are hydrated and simply entangled with one another. As temperature increases, macromolecules gradually lose their water of hydration, until polymer-polymer hydrophobic associations take place, thus forming the hydrogel network. The sol-gel transition temperature depends on the degree of substitution of the cellulose ethers as well as on the addition of salts. A higher degree of substitution of the cellulose derivatives provides them a more hydrophobic character, thus lowering the transition temperature at which hydrophobic associations take place. A similar effect is obtained by adding salts to the polymer solution, since salts reduce the hydration level of macromolecules by recalling the presence of water molecules around themselves. Both the degree of substitution and the salt concentration can be properly adjusted to obtain specific formulations gelling at 37 °C and thus potentially useful for biomedical applications [33,34,35]. Liquid formulations, either mixed with therapeutic agents or not, are envisaged to be injected *in vivo* and their crosslinking reaction triggered by the only physiological environment. However, physically crosslinked hydrogels are reversible [36], thus might flow under given conditions (*e.g.*, mechanical loading) and might degrade in an uncontrollable manner. Due to such drawbacks, physical hydrogels based on MC and HPMC are not recommended for use *in vivo*. *In vitro*, MC hydrogels have been recently proposed as novel cell sheet harvest systems [34].

As opposed to physical hydrogels which show flow properties, stable and stiff networks of cellulose can be prepared by inducing the formation of chemical, irreversible crosslinks among the cellulose chains. Either chemical agents or physical treatments (*i.e.*, high-energy radiation) can be used to form stable cellulose-based networks. The degree of crosslinking, defined as the number of crosslinking sites per unit volume of the polymer network, affects the diffusive, mechanical and degradation properties of the hydrogel and can be controlled to a certain extent during the synthesis. Specific chemical modifications of the cellulose backbone might be performed before crosslinking, in order to obtain stable hydrogels with given properties. For instance, silylated HPMC has been developed which crosslinks through condensation reactions upon a decrease of the pH in water solutions. Such hydrogels show potential for the *in vivo* delivery of chondrocytes in cartilage tissue engineering [37,38]. As a further example, tyramine-modified sodium carboxymethyl cellulose (NaCMC) has been synthesized to obtain enzimatically gellable formulations for cell delivery [39]. Photocrosslinking of water solutions of cellulose derivatives is achievable following proper functionalization of cellulose.

Depending on the cellulose derivatives used, a number of crosslinking agents and catalysts can be employed to form hydrogels. Epichlorhydrin, aldehydes and aldehyde-based reagents, urea derivatives, carbodiimides and multifunctional carboxylic acids are the most widely used crosslinkers for cellulose. However, some reagents, such as aldehydes, are highly toxic in their unreacted state. Although unreacted chemicals are usually eliminated after crosslinking through extensive washing in distilled water, as a rule toxic crosslinkers should be avoided, in order to preserve the biocompatibility of the final hydrogel, as well as to ensure an environmentally sustainable production process. The crosslinking reactions among the cellulose chains activated by chemical agents might take place in water solution, organic solvents or even in the dry state (*e.g.*, polycarboxylic acids can crosslink cellulose macromolecules via condensation reactions which are favored at high temperature and in the absence of water [40,41,42,43]). Novel superabsorbent cellulose-based hydrogels crosslinked with citric acid have been recently reported, which combine good swelling properties with biodegradability and absolute safety of the production process [43].

In light of environmental and health safety concerns, radiation crosslinking of polymers, based on gamma radiation or electron beams, has been receiving increasing attention in the last years as it does not involve additional chemical reagents, is easily controllable and, in cases of biomedical applications, allows the simultaneous sterilization of the product. High-energy radiation usually leads to chain scission of the polymer and this has been shown also for cellulose [44]. However, several cellulosics can be crosslinked (*i.e.*, crosslinking prevails over degradation) under relatively mild radiation, both in aqueous solutions and solid form [45,46,47]. The crosslinking reaction is affected by the irradiation dose as well as by the cellulose concentration in solution.

## 3. Applications of Cellulose-based Hydrogels

In cases where biodegradability of a hydrogel is required or recommended, cellulosics are appealing hydrogel precursor materials, due to their low cost, the large availability and biocompatibility of cellulose, and the responsiveness of some cellulosics to variations of external stimuli. This section deals with some of the possible applications of cellulose-based hydrogels, which range from the traditional use of hydrogels as water absorbents to more innovative biomedical applications.

### 3.1. Superabsorbents for Personal Hygiene Products

Acrylate-based superabsorbent hydrogels are currently extensively used in personal care products to absorb fluids. They keep moisture and wetness away from the skin, which helps promoting skin health and consumer comfort. It is estimated that the majority of parents in industrialized countries, along with thousands of hospitals and group care centers around the world, use disposable diapers containing a superabsorbent polymer (SAP). In addition, there is an increasing use of this material in training pants and adult incontinence products worldwide. A number of studies have been published in recent years documenting the advantages resulting from the use of superabsorbent materials in personal care products and their safety and effectiveness [48,49]. In addition to keeping skin dry and preventing diaper rash, the SAP helps control the spread of germs in group care settings. The leakage prevention aspect of diapers is very important for reducing the risk of fecal contamination in day care play areas and thus the potential for the spread of illness [50]. Several medical studies have provided clear evidence that disposable diapers play an important role in reducing the risk of spread of gastrointestinal illnesses and are significantly more effective than double cloth diapers and plastic overpants [51].

**Figure 2:**
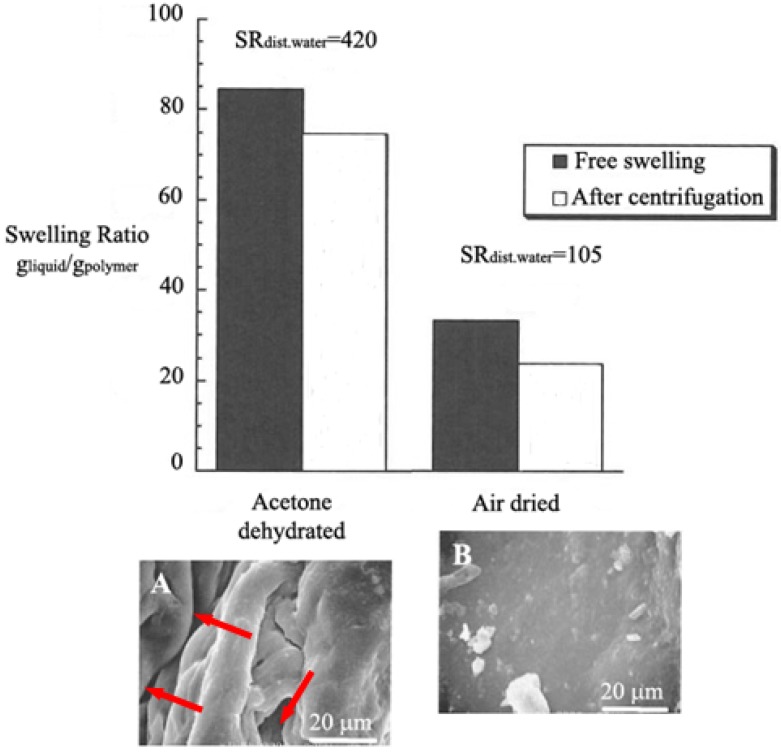
Average uptake of synthetic urine by NaCMC/HEC hydrogels, both in free swelling conditions and after centrifugation [56]. The swelling ratio SR is provided for acetone-dehydrated hydrogels (SEM micrograph A: red arrows indicate the voids or micropores), and air-dried ones (SEM micrograph B: smooth surface with no pores; exogenous white particles are likely due to air drying and manipulation). The values of SR in distilled water are also provided (n=5).

Although Harper [52] and Harmon [53] separately filed their patents for superabsorbents in 1966, the first use of superabsorbents in the diaper industry was only introduced in 1982 by Unicharm in Japan, following its use in sanitary napkins. With the superabsorbent materials a new generation of high performance diapers was created. Not only did the diapers become thinner but also they had improved retention performance which helped reduce leakage and diaper rash [54]. Premium diapers with leakage values below 2% became a reality. The average weight of a typical medium size diaper was reduced by about 50%. This was advantageous in terms of environmental issues, and it also made good economical sense due to the reduced packaging cost. With regard to the current ecological impact of disposable diapers and other similar articles, it is worth providing a brief estimate of diaper consumption. A child till the age of 30 months uses approximately six disposable diapers a day. Each of the diapers has an average volume of 500 cc, so one child produces on average 3,000 cc of garbage a day, *i.e.* 1,092 cubic meters per year. Since there are about 50,000 diaper users per million population, every day it would be necessary to remove 150 cubic meters of diapers from a city of one million inhabitants. Different attempts have been made to recycle disposable diapers, napkins, hospital bed sheets, sanitary towels and other similar products [55]. All these cellulose containing articles have the same structure: an envelope of non woven tissue, a plastic cover material, and an absorbent fluff of wood pulp cellulose, mixed in most cases with SAP. The basic idea of diaper recycling is to recover separately the cellulose, which is biodegradable and recyclable, the plastic cover material and the SAP, both of which are not biodegradable but might be recycled for other uses. The complexity of such a process has prompted the parallel development of biodegradable diapers, *i.e.* possessing a biodegradable plastic cover, which, however, still contain the non-degradable acrylate-based SAP. An alternative solution to the problem of SAP recycling has been recently suggested, and has been envisaged in the use of cellulose-based hydrogels, which are totally biodegradable. Novel hydrogels, based on sodium carboxymethylcellulose (NaCMC) and hydroxyethyl cellulose (HEC) crosslinked with DVS, possess swelling capabilities comparable with those displayed by SAP, and high water retention capacities under centrifugal loads [56] (Figure 2). These significant results have been achieved by inducing a microporous structure in the hydrogel, by means of a phase inversion desiccation technique in acetone (*i.e.*, a non-solvent for cellulose), which increased the water absorption, as well as the swelling kinetics, due to capillarity effects. When comparing the scanning electron microscopy (SEM) images of an acetone-dehydrated *vs.* an air-dried cellulose-based hydrogel (Figure 2), the surface of the former displays foldings and voids, whereas the latter shows a smooth and dense surface.

The main advantage displayed by cellulose-based hydrogels over current SAP lies in their biodegradability and environmentally friendly nature. In this context, recent innovations about the cellulose-based hydrogels described above regard the implementation of an environmentally sustainable production process [57], and the use of non-toxic crosslinking agents [25,43]. Cellulose radiation crosslinking, which does not require the use of further chemicals, might be of value in the development of novel environmentally friendly superabsorbents.

### 3.2. Water Reservoirs in Agriculture

There is an increasing interest in using superabsorbent hydrogels in agriculture. This is mainly due to the need to reduce water consumption and optimize water resources in agriculture and horticulture, and has a role in the promotion of a novel approach of human habit and culture towards water, to be treated as a benefit to save and not as an excess to waste.

**Figure 3:**
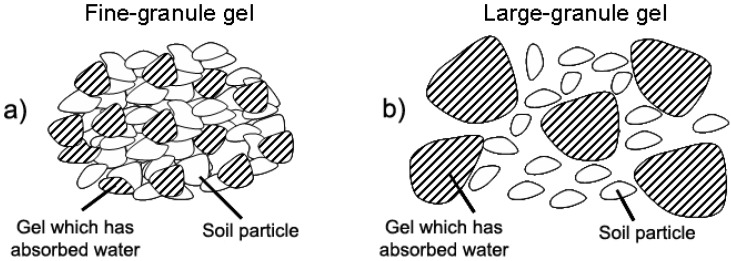
(a) When mixing the soil with a fine-granule gel, air flow is limited and a layer of substrate/gel mixture might form, which further limits the flows of air and water within the soil. (b) With a large-granule gel, a better air flow through the soil is yielded, resulting in higher oxygenation to plant roots. Pictures (a) and (b) represent only one of the possible spatial distributions of hydrogel and soil particles.

During the swelling process of a hydrogel, it is well known that the material turns from a glassy to a rubber-like state, which is able to store water even under significant compression. However, the swollen hydrogel can slowly release water through a diffusion-driven mechanism, if a gradient of humidity between the inside and the outside of the material exists. Along with water, further molecules, loaded in the polymer network during its synthesis, can be released in a controlled and sustained manner by means of diffusion. With the aim of making cultivations possible in arid and desert regions of the world, where scarcity of water resources is a relevant issue, the xerogel (*i.e.*, dry hydrogel), in form of powder or granules, is envisaged to be mixed to the soil, in the area close to plant roots. The xerogel might also be loaded with nutrients and/or plant pharmaceuticals. As the cultivation is watered, the water is absorbed by the hydrogel, which then releases water and nutrients to the soil as needed, thus keeping the soil humid over long periods of time. This process allows a high saving of water, which is not lost soon after the watering due to evaporation and drainage, and a re-distribution of the water resources available for cultivation in other applications. A further advantage in using hydrogels in this application is related to the effect of the swelling itself on the soil. Indeed the hydrogel granules, which in the dry form have almost the same dimensions of the substrate granules, increase their dimension after swelling, thus increasing soil porosity and providing a better oxygenation to the plant roots (Figure 3). This also suggests that large-granule hydrogels are likely to yield better results than fine-granule ones, if properly mixed with the soil (indeed different spatial configurations for the soil and hydrogel particles are possible, depending on their densities and the soil-hydrogel and hydrogel-hydrogel interactions).

There are a number of commercially available products able to absorb, retain and release water to the soil. It has been demonstrated that all these products are effective in saving water. However, due to their extremely high water retention capacity, over-dosage of such products is potentially dangerous to cultivations and should be avoided. A few research studies have been carried out to determine appropriate hydrogel amounts and application rates for different environmental conditions and different plant species [58]. It is worth noting that, being acrylate-based, most commercial products are not biodegradable. Cellulose-based hydrogels fit perfectly in the current trend to develope environmentally friendly alternatives to acrylate-based superabsorbent hydrogels. Sannino and co-workers recently developed a novel class of totally biodegradable and biocompatible microporous cellulose-based superabsorbent hydrogels [29,56,59,60]. Such hydrogels are able to absorb up to one liter of water per gram of dry material, without releasing it under compression. The hydrogel can be produced both in form of powder or of a bulky material with a well defined shape and a strong memory of its shape after swelling. The material can be charged with small molecules, such as nutrients, to be released under a controlled kinetic. Among the possible applications of this material, a study has been performed on its specific use as water reservoir in agriculture. Hydrogel sorption capacity has been tested at different ionic strength of the swelling medium, with the aim to simulate as much as possible the hydrogel-soil interaction. To prove the feasibility and the efficacy of the proposed technology, a pilot scale production plant has been developed to produce the amount of hydrogel necessary for some studies being carried out in experimental greenhouses in the South of Italy, where the paucity of water is a common problem. Preliminary results show great promise. The soil with the addition of small quantities of the product is able to remain humid for periods more than four times longer with respect to the soil watered without the presence of the hydrogel.

### 3.3. Body Water Retainers

Due to the intrinsic biocompatibility of cellulose, together with the biocompatibility and the versatile properties displayed by hydrogels in biomedical applications, cellulose-based hydrogels are appealing materials for a number of applications *in vivo*. For example, hydrogels hold promise as devices for the removal of excess water from the body, in the treatment of some pathological conditions, such as renal failure and diuretic-resistant oedemas. The hydrogel in powder form is envisaged to be administered orally and absorb water in its passage through the intestine, where the pH is about 6-7, without previously swelling in the acid environment of the stomach. The hydrogel is then expelled through the foecal way, thus performing its function without interfering with body functions. As sensitivity to pH is required, polyelectrolyte cellulose hydrogels, based on NaCMC and HEC, have been investigated for such applications [29,60,61]. The hydrogels showed good swelling properties at neutral pH and low swelling ratios at acid pHs. Moreover, the water sorption capability could be modulated and maximized by adjusting the ratio NaCMC/HEC and the amount of crosslinker used (divynilsulphone, DVS) [29,60], and by adding molecular spacers to the polymer network, *e.g.* polyethylene glycol (PEG) [61]. In spite of the use of DVS as a crosslinker, the hydrogel formulations tested showed a good biocompatibility both in *in vitro* and *in vivo* experiments. The use of hydrogels in combination with diuretic therapies might be useful in substituting some drugs and in using an intestinal pathway, instead of the systemic one, to remove water from the body [60].

### 3.4. Stomach Bulking Agents

The latest World Health Organization projections indicate that at least one in three of the world's adult population is overweight and almost one in 10 is obese. Additionally there are over 20 million children under age five who are overweight. Obesity and overweight represent the second cause of death after smoking, as major risk factors for several chronic diseases, such as type 2 diabetes, cardiovascular disease, sleep apnea, hypertension, stroke, and certain forms of cancer. Moreover, being overweight or obese often has a dramatic impact on the psychological well-being, reducing the overall quality of life [62,63,64]. The treatment of overweight and obesity usually consists of a supervised diet, often combined with adequate physical exercise. In the most serious cases, surgical procedures, that involve essentially gastric restriction, or particular drug treatments, may be required [62]. However, in the recent years, a number of dietary supplements and meal replacements have been developed and sold as over-the-counter slimming aids. Dietary supplements are claimed to act either by binding fats and so reducing fat absorption, as reported for chitosan-based products, or by directly reducing the appetite, as for different natural fibers and herbal products, that seem to absorb liquids and swell inside the stomach, thus giving a sense of fullness [65,66].

The latter approach, based on the use of natural fillers or bulking agents, is very interesting for its great potential of reducing the amount of food intake by reducing the available space in the stomach, without the need of complex surgical interventions. However, there is no clear evidence of the effectiveness of currently available bulking agents in promoting weight loss, neither in the short-term nor in the long-term, whereas their adverse effects, usually including gastrointestinal symptoms, have been well documented [65,66]. Moreover, it should be taken into account that some fillers may be harmful, causing obstructions in the intestines, stomach, or esophagus, as reported for guar gum [67]. Therefore, the development of novel bulking agents, effective in promoting weight loss, is needed. In this direction, superabsorbent hydrogels are of particular interest, since not only can their swelling capacity be properly designed by controlling their chemical composition and physical microstructure, but it can also be modulated by changing the environmental conditions (*e.g.*, pH, ionic strength, temperature). The basic idea is that a xerogel-based pill is administered orally before each meal, and that the xerogel powder swells, once in the stomach. In such a way, the space available for food intake is reduced, giving a feeling of fullness. The swollen hydrogel is then eliminated from the body by foecal way. In this perspective, the hydrogel is envisaged to pass through the gastrointestinal tract, thus it is supposed to encounter the different pH environments of the stomach and the intestine. Along with superporous acrylate-based hydrogels, which swell very rapidly in aqueous solutions [68], novel cellulose-based hydrogels, obtained by crosslinking aqueous mixtures of NaCMC and HEC, have been shown to be appealing for the production of dietary bulking agents [25,69]. Indeed such hydrogels possess a high biocompatibility, with respect to intestinal tissues, and a high, pH-sensitive water retention capacity [69]. Although the polyanionic nature of the CMCNa network provides higher swelling capabilities at neutral pHs rather than at acid ones, the swelling ratio obtained at acid pHs might still be significant for use of the hydrogel as stomach filler. In particular, cellulose-based hydrogels obtained from non-toxic crosslinking agents are particularly attractive for this kind of application [25,43,69].

### 3.5. Devices for Controlled Drug Delivery

Cellulose ethers have long been used in the pharmaceutical industry as excipients in many drug device formulations [70]. Their use in solid tablets allows a swelling-driven release of the drug as physiological fluids come into contact with the tablet itself. The cellulose ether on the tablet surface (*e.g.*, HPMC) starts to swell, forming chain entanglements and a physical hydrogel. As swelling proceeds from the swollen surface to the glassy core of the tablet, the drug progressively dissolves in water and diffuse out from the polymer network. The rate of drug release depends on the water content of the swollen hydrogel, as well as on its network parameters, *i.e.*, degree of crosslinking and mesh size [4,71]. Depending on the structure of the particular cellulose ether used, chain dissolution may take place along with swelling due to the physical nature of the hydrogel network, thus drug release results from the complex combination of swelling, diffusion and erosion mechanisms.

**Figure 4:**
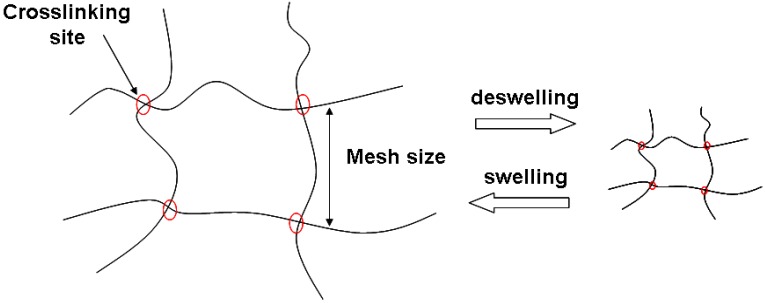
Schematic structure of a tetrafunctional polymer network upon swelling/deswelling transitions. What changes is the mesh size of the network, which determines the free space available for diffusion and thus regulates the diffusion of molecules (*e.g.*, drugs) through the network itself.

More sophisticated hydrogel-based devices other than swelling tablets have been developed for controlled drug delivery. The most recent advances aim not only at the sustained release of a bioactive molecule over a long time period, ranging from hours to weeks, but also at a space-controlled delivery, directly at the site of interest. The need to encapsulate bioactive molecules into a hydrogel matrix or other delivery devices (*e.g.*, microspheres) is also related to the short half-life displayed by many biomolecules *in vivo*. When using hydrogels to modulate the drug release, the loading of the drug is performed either after crosslinking or simultaneously, during network formation [14]. Moreover, the bioactive molecule can be covalently or physically linked to the polymer network, to further tune the release rate. Smart hydrogels are particularly useful to control the time- and space-release profile of the drug, as swelling-deswelling transitions, which modify the mesh size of the hydrogel network (Figure 4), occur upon changes of physiologically relevant variables, such as pH, temperature and ionic strength [4].

Controlled release through oral drug delivery is usually based on the strong pH variations encountered when transitioning from the stomach to the intestine. Cellulose-based polyelectrolyte hydrogels (*e.g.*, hydrogels containing NaCMC) are particularly suitable for this application. For instance, anionic hydrogels based on carboxymethyl cellulose have been investigated recently for colon-targeted drug delivery [72].

The most recent advances in controlled release through a hydrogel matrix deal with the delivery of proteins, growth factors and genes to specific sites, the need for which has been prompted by tissue engineering strategies. While hydrogel formulations for oral and transdermal delivery can be non-degradable, the direct delivery of drugs or proteins to different body sites requires the hydrogel biodegradation, in order to avoid foreign body reactions and further surgical removal. Injectable hydrogel formulations are particularly appealing and currently under investigation. The crosslinking reaction has to be performed under mild conditions in order not to denaturate the loaded molecule. The microenvironment resulting from degradation of the polymer should be mild as well to the same purpose. With particular regard to cellulose-based hydrogels, injectable formulations, based on HPMC, have been developed to deliver both biomolecules and exogenous cells *in vivo* [37,38,73].

### 3.6. Scaffolds for Regenerative Medicine

Regenerative medicine is an interdisciplinary field which deals with the induced regeneration of tissues and organs *in vivo*, by means of a scaffolding material or template that guide and support the cells during the synthesis of new tissues. Due to their large water content, hydrogels are highly biocompatible, possess rubbery mechanical properties close to those of soft tissues and usually allow the incorporation of cells and bioactive molecules during the gelling [14,74]. Moreover, although cells do not readily attach to highly hydrophilic surfaces, the bulk or surface chemistry of hydrogels can be easily modified with extracellular matrix (ECM) domains, which promote cell adhesion as well as specific cell functions [15]. Hydrogels are thus likely to be ideal platforms for the design of biomimetic scaffolds for tissue regeneration.

In the last decade, the use of cellulose and its derivatives as biomaterials for the design of tissue engineering scaffolds has received increasing attention, due to the excellent biocompatibility of cellulose and its good mechanical properties. Ideally, for optimal tissue regeneration, the scaffold should be biodegradable, with a biodegradation rate matching that of the biological process of interest, but practically a slow degradation is often preferred, in order to minimize the risks associated to a premature resorption of the scaffold. In spite of its biodurability, cellulose is thus a candidate biomaterial for the design of tissue engineering scaffolds. Nonetheless, it is well known that a biodurable material or a too slow degradation may lead to undesired biological responses (*e.g.*, a foreign body reaction) in the long term, which limit the possible applications of cellulose in regenerative medicine. Cellulose and its derivatives, usually in the form of sponges or fabrics, have been applied so far for the treatment of severe skin burns, and in studies on the regeneration of cardiac, vascular, neural, cartilage and bone tissues [21,35,37,38,73,75,76,77,78]. With particular regard to cellulose-based hydrogels, a few independent investigations show that cellulose-based hydrogels are potentially useful for inducing the regeneration of bone, cartilage and neural tissues [35,38,78,79]. In a recent study, the pre-treatment of a cellulose-based scaffold with cellulase prior to implantation has been proposed, in an attempt to modulate the *in vivo* degradation behaviour of cellulose [21]. The authors also pointed out that the final product of cellulose degradation is glucose, which is a nutrient for cells, thus underscoring the advantage in using cellulose rather than other synthetic or natural polymers for tissue engineering applications.

**Figure 5:**
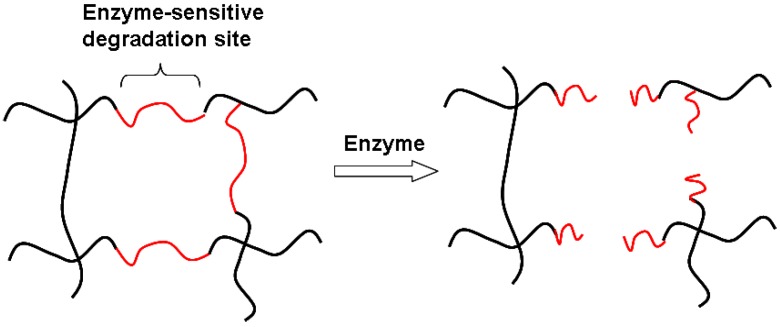
***Invivo*** degradation of a polymer network (*e.g.*, cellulose hydrogel) functionalized with enzyme-sensitive moieties (*e.g.*, hyaluronic acid). The degradation rate is dependent on the amount of degradable sites as well as the degree of crosslinking of the network, which affects enzyme diffusivity through the mesh size.

While the addition of biomimetic domains to cellulose has been performed to enhance cell adhesion [35,75,76,78,80,81,82], the functionalization of cellulose with ECM moieties that may tune its biodegradation rate has not been widely investigated. Cellulose-based hydrogels are advantageous over cellulose sponges and fabrics as their bulk chemistry can be easily modified. Independent investigations have reported the synthesis of novel biomimetic hydrogels, based on crosslinking cellulose derivatives with hyaluronic acid (HA) [24,25,26]. Although mainly proposed as post-operative adhesion barriers, such hydrogels show also potential as scaffolds for regenerative medicine, with a tunable degradation rate. Indeed the presence of HA in the cellulose network provides enzyme-sensitive degradation sites, whose density in the bulk of the hydrogel can be easily controlled (Figure 5).

The biocompatibility of the crosslinking agent used is particularly important, especially in cases where reactive groups of the crosslinker are incorporated into the hydrogel network and might then be released upon degradation. Hydrogels based on HEC, NaCMC and HA have been recently crosslinked with a water-soluble carbodiimide, which is both non-toxic and a ‘zero-length’ crosslinker [25]. The carbodiimide, which is washed out from the polymer network after the synthesis, is well-known to induce the formation of ester bonds among polysaccharide molecules without taking part in the linkage [83]. It is worth noting that the ester bonds forming the network have the potential to be digested, in the long term, via hydrolysis, as it normally occurs for synthetic polyesters. Thus cellulose-based hydrogels crosslinked with carbodiimide show potential for a tunable biodegradation rate, even when not containing HA. Although an investigation on the biodegradation behaviour of such hydrogels has not been performed yet, it is reasonable to hypothesize that the degradation rate can be modulated to some extent by controlling the degree of crosslinking. The mechanical stiffness of the hydrogel is also dependent on the degree of crosslinking and should be designed according to the tissues being addressed. Furthermore, the carbodiimide-mediated crosslinking reaction holds promise for the functionalization of cellulose with several biomolecules, able to promote specific cell functions, due to the ability of the carbodiimide to crosslink various polypeptides [84,85]. This opens a wide range of possibilities for the design of biomimetic, cellulose-based hydrogel scaffolds for tissue engineering.

A final remark regarding the development of regenerative templates concerns the key role played by the scaffold porosity, which enhances the attachment, infiltration and survival of cells within the scaffold. Due to their nano-sized mesh structure [71], hydrogels are usually employed to treat small tissue defects, while fail in larger implants. Novel manufacturing techniques aiming at the development of several types of porous hydrogels are being investigated [86,87,88,89], and might be of great value in enhancing the regenerative potential of cellulose-based hydrogels as well.

### 3.7. Wound Dressings

Inflammation, autolytic debridement, granulation tissue formation and re-epithelialization are the processes which normally occur during wound healing. Appropriate wound dressings are designed to promote healing while protecting the wound from infection. This is particularly important in cases of chronic wounds (*e.g.*, ulcers), which fail to heal properly. Since a moist environment encourages rapid healing [90], hydrogels are optimal candidates for the development of wound dressings, either as sheets or in amorphous form. Amorphous hydrogels are usually physically crosslinked, thus their viscosity decreases as they absorb physiological fluids. Such gels may be packaged in tubes or in foil packets, and in the latter case the gels are reinforced with a gauze or a polymeric mesh to allow an easy removal and prevent gel liquefaction. Hydrogels should be designed to maintain the right moisture balance in the wound bed, by hydrating the wound surface and/or absorbing the wound exudates. They also provide non-adherent dressings which can be easily removed without any damage to the wound bed. Hydrogel transparency is a further advantage in this application, as wound healing can be easily monitored. Various types of hydrogel dressings have been patented so far and are commercially available, based on synthetic or natural polymers, or a combination of them. Among the most recent patents, it is worth citing those describing *in situ* forming gels (*e.g.*, based on sprayable formulations [91] and on coalescing nanoparticles [92]), and those exploring radiation crosslinking as a stabilization tecnique, which allows to obtain sterile and crosslinked hydrogel films in a single-step process [93,94]. The most advanced hydrogel dressings include antimicrobial agents, such as silver ions, in their formulation [94].

Due to its purity and high water retention capacity, bacterial cellulose (BC) has been widely investigated for wound healing and a series of BC-based wound dressings are currently marketed [18]. To the best of our knowledge, gel-forming cellulose derivatives, such as NaCMC, are included in the formulation of some commercially available hydrogel dressings, usually in combination with propylene glycol, which works as a humectant and a preservative (Table 1). It is worth noting that the products developed so far are usually indicated for the treatment of specific wounds and often require the use of secondary dressings. Therefore, a number of investigations currently deal with the development of novel wound dressings with improved performance, and cellulose-based hydrogels appear to be promising candidates. Preliminary, unpublished results by the present authors show that cellulose-based hydrogels crosslinked with hyaluronic acid [25] induce a good proliferation of keratinocytes, following a scratch wound model in *in vitro* culture.

**Table 1:** Commercially available hydrogel wound dressings containing carboxymethylcellulose (CMC) or sodium CMC (NaCMC). Most dressings are available in two forms, either as sheets or as amorphous gels. Products containing silver ions show antimicrobial activity.

Hydrogel wound dressing (Producer)	Composition
IntraSite^TM^ Gel (Smith and Nephew)	Water
	Propylene glycol
	NaCMC
GranuGel^TM^ (ConvaTec)	Water
	NaCMC
	Propylene glycol
	Pectin
Purilon Gel^TM^ (ColoPlast)	Water
	CMC
	Calcium alginate
Aquacel Ag^TM^	NaCMC
(ConvaTec)	Silver ions (1.2%)
Silvercel^TM^ (Johnson & Johnson)	Calcium alginate
	CMC
	Silver ions (8%)

## 4. Conclusions

Cellulose-based hydrogels are biocompatible and biodegradable materials which show promise for a number of industrial uses, especially in cases where environmental issues are concerned, as well as biomedical applications. Several water-soluble cellulose derivatives can be used, singularly or in combination, to form hydrogel networks possessing specific properties in terms of swelling capability and sensitivity to external stimuli. The current trend in the design of cellulose hydrogels is related to the use of non-toxic crosslinking agents or crosslinking treatments, to further improve the safety of both the final product and the manufacturing process. The smart behaviour of some cellulose derivatives (*e.g.*, NaCMC, HPMC) in response to physiologically relevant variables (*i.e.*, pH, ionic strength, temperature) makes the resulting hydrogels particularly appealing for *in vivo* applications. In spite of the non-bioresorbability of cellulose, the possibility to functionalize cellulose-based hydrogels with bioactive and biodegradable extracellular matrix domains suggests that, in the near future, such hydrogels might be ideal platforms for the design of scaffolding biomaterials in the field of tissue engineering and regenerative medicine.
